# Clinician–patient communication: evidence-based recommendations to guide practice in cancer

**DOI:** 10.3747/co.v16i6.432

**Published:** 2009-12

**Authors:** G. Rodin, C. Zimmermann, C. Mayer, D. Howell, M. Katz, J. Sussman, J.A. Mackay, M. Brouwers

**Affiliations:** * Department of Psychosocial Oncology and Palliative Care, Princess Margaret Hospital, University Health Network, Toronto, ON; † Palliative Care Services, Princess Margaret Hospital, University Health Network, Toronto, ON; ‡ Supportive Care Program, Supportive Care Oncology Research Unit and Sudbury Genetic Counselling Services, Regional Cancer Program of the Hôpital Régional de Sudbury Regional Hospital, Sudbury, ON; § Oncology Nursing Research, Princess Margaret Hospital, University Health Network, Toronto, ON; || Southlake Regional Health Centre, Newmarket, ON; # Supportive Cancer Care Research Unit, McMaster University, Hamilton, ON; ** Health Information Research Unit, Faculty of Health Sciences, McMaster University, Hamilton, ON; †† McMaster University and Cancer Care Ontario Program in Evidence-Based Care, Hamilton, ON

**Keywords:** Practice guideline, systematic review, cancer, communication, relationships

## Abstract

**Goals of Work:**

To develop recommendations for effective communication between cancer health care providers and patients based on a systematic review of methods of clinician–patient communication that may affect patient outcomes associated with distress at critical points in the course of cancer care.

**Materials and Methods:**

A systematic review of the literature was conducted, and evidence-based recommendations were formulated to guide clinician–patient communication in cancer care. A formal external review was conducted to validate the relevance of these recommendations.

**Main Results:**

Recommendations for communication in cancer care are presented, based on

- guidelines from the Australian National Breast Cancer Centre and the Australian National Cancer Control Initiative,
- an updated systematic review of the research evidence, and
- a consensus by the Clinician–Patient Communications Working Panel of the Program in Evidence-Based Care of Cancer Care Ontario.

The recommendations were sent to 110 Ontario practitioners for external review: 33 responded (30% response rate). Most of these respondents (87%) agreed with the draft recommendations and approved of their use as a practice guideline (90%). A condensed version of the recommendations, including 10 key points, was also created.

**Conclusions:**

There is evidence to support general clinician–patient communication approaches, although the preferences of cancer patients regarding such communication exhibit individual and cultural variability. Recommendations are provided, based on evidence, the consensus of an expert panel, and feedback from a survey of external practitioners. Evidence evaluating the role of decision aids and strategies to facilitate better communication is inconsistent, although such tools may be of value for some patients.

## 1. INTRODUCTION

A relationship of health care providers with patients, based on trust, respect, and reciprocity, and with mutually negotiated goals and expectations, can be an important support and buffer for cancer patients experiencing distress. Communication serves to build and maintain this relationship, to transmit information, to provide support, and to negotiate treatment decisions. The process of communication between providers and cancer patients can significantly affect clinical outcomes^1^. However, recent evidence demonstrates that communication in cancer care may often be suboptimal, particularly with regard to the emotional experience of the patient^2^. The aim of the present paper is to present recommendations for effective clinician–patient cancer-related communication during consultation visits, which may prevent or alleviate patient distress. We hope that the recommendations will help to promote empathic and effective communication in medical and cancer care settings.

Cancer Care Ontario’s Program in Evidence-Based Care (pebc) is a provincial initiative responsible for developing evidence-based clinical practice guidelines (cpgs) in Ontario. The pebc guidelines are used to facilitate effective practice, to guide provincial and institutional policy, and to enable access to treatments in the publicly funded provincial health care system^3^–^5^. The pebc is one component of a larger formalized cancer system defined by data about, and monitoring of, system performance; evidence-based knowledge and best practices; transfer and exchange of such knowledge; and strategies to leverage implementation of knowledge. Methods used by the pebc include the systematic review of research evidence by teams comprising clinical, content, and methodology experts; interpretation of, and team consensus about, the evidence; development of recommendations; and formal standardized external review of all draft cpgs^3^,^4^,^6^–^9^. The external review process involves disseminating draft cpgs and a validated survey, Clinician’s Assessments of Practice Guidelines in Oncology, to a sample of clinicians for whom the cpg is relevant^6^. To create an appropriate sample, defining features of the cpg (topic, modality of care, disease site) are matched with professional characteristics of clinicians and content experts held in a comprehensive database of clinicians involved in cancer care in the province. Reminders are sent to non-responders at 2 weeks (postcard) and at 4 weeks (second full package), with closure of the review process typically occurring between weeks 7 and 8.

Using the foregoing methods, the clinical recommendations presented here were developed by the Clinician–Patient Communications Working Panel of the pebc of Cancer Care Ontario. The recommendations are based on a systematic review of cancer-related communication, which is available in a companion document^10^. This systematic review is a convenient source of the best evidence currently available on clinician–patient communication relating to critical points of care (cancer diagnosis, recurrence or metastatic disease, and end of life) and its effect on patient distress. The recommendations are intended to promote evidence-based practice and to improve quality of care for patients.

## 2. MATERIALS AND METHODS

### 2.1 Systematic Review

The systematic review was conducted by the Clinician–Patient Communications Working Panel of the pebc^3^,^4^. The methods are described in detail in another publication^10^. Evidence-based practice guidelines with explicit literature selection criteria, systematic reviews, meta-analyses, and randomized trials were all eligible for the review. The quality of the guidelines, randomized trials, and systematic reviews was assessed using standardized criteria for each type of publication.

### 2.2 Development of Evidence-Based Recommendations

After the systematic review, the Clinician–Patient Communications Working Panel developed evidence-based recommendations. The review was completed in 2007, and the guidelines were completed in 2008. A decision was made to use the Australian National Breast Cancer Centre and the Australian National Cancer Control Initiative (nbcc-ncci), Clinical Practice Guidelines for the Psychosocial Care of Adults with Cancer^11^ as a framework for the recommendations, supplemented by additional information from the systematic review. Based on this framework, general evidence-based recommendations are provided first, followed by more specific recommendations related to the stage in the diagnostic or treatment process.

The recommendations developed by the Working Panel were sent to Ontario cancer practitioners for external review. Practitioner feedback was obtained through a mailed survey of 110 participants in Ontario, including nurses, social workers, patient educators, palliative care physicians, psychiatrists, psychologists, oncologists, surgeons, and family physicians. The external review group also included members of the Provincial Palliative Care Committee and the Provincial Psychosocial Oncology Committee. The survey items asked respondents to evaluate the methods, results, and discussion used to inform the draft recommendations. Respondents were then asked to indicate whether the draft recommendations should be approved as a guideline. Written comments were invited. The Clinician–Patient Communications Working Panel reviewed the results of the survey, and consensus in this expert group was achieved by teleconference discussion. There was no instance in which external consultation was needed to achieve consensus.

## 3. RESULTS

### 3.1 External Review

From among the 110 surveys sent, 33 responses were received (30% response rate). Responses include returned completed surveys, and telephone, fax, and e-mail responses. Of the respondents, 60% were nurses; 17%, surgeons; 11%, family physicians; 6%, medical oncologists; and 3% each, patient educators and medical physicists. Of the participants who responded, 31 (94%) indicated that the report was relevant to their practice or organizational position, and of those 31, 26 (84%) agreed with the summary of evidence, 27 (87%) agreed with the recommendations, and 28 (90%) approved of the recommendations as a practice guideline.

### 3.2 Summary of Main Findings

Almost all respondents agreed with the recommendations, and 20 respondents (61%) provided written comments, some suggesting changes to the guideline. Several respondents remarked that professional translation services and not hospital staff, family, or volunteers should be used to translate important information during clinical interviews. Others suggested that more information should be included regarding methods to address the differences in communication preferences between patients from various cultures. One respondent recommended that the guideline should include having a conversation about disease progression before the transition to end-of-life care begins. And one practitioner commented that a condensed version of the recommendations would be useful.

### 3.3 Modifications and Actions by the Clinician– Patient Communications Working Panel

The nbcc-ncci *Clinical Practice Guidelines for the Psychosocial Care of Adults with Cancer* were selected as a framework for this area of practice. The recommendations that follow reflect integration of the nbcc-ncci recommendations, an updated systematic review of research evidence since the release of those guidelines, and consensus among the Clinician–Patient Communications Working Panel members. Based on the comments obtained from practitioners during the external review, recommendations were added suggesting that the provider consider the patient’s specific cultural needs and preferences, use professional translation services when possible, and consider the patient’s ability to communicate in English. As well, the term “end of life” was changed to “disease progression” to reflect the request to begin conversations before end-of-life care begins. Furthermore, a 10-point summary of important elements to consider when communicating with patients was created.

## 4. RECOMMENDATIONS

The recommendations that follow were based on evidence that met nbcc-ncci evidence grading levels i and ii—that is, they were obtained from systematic reviews of all relevant randomized controlled trials (rcts) or from at least one properly designed rct. This evidence-based rationale for the recommendations is summarized in each of the sections, followed by the specific recommendations that flow from that rationale. The focus in these recommendations is on the process of communication at specific stages in the disease and treatment trajectory, rather than on the content of the communication.

### 4.1 General Interaction Skills

#### 4.1.1 Evidence-Based Rationale

The way in which clinicians and the treatment team relate to and communicate with patients can benefit those patients and their families by improving aspects of psychosocial adjustment, decision-making, treatment compliance, and satisfaction with care.

Aspects of communication that are important include empathic listening and creating an environment in which patients are free to express feelings and ask questions.

Understanding, recall, and satisfaction with care may increase when techniques are used to reinforce or record what has been communicated to patients. These techniques may include taping consultations, providing general information tapes, sending summary letters as a follow-up to consultation, and including a support person in the clinical visit.

#### 4.1.2 Recommended Approach: General Interaction Skills

The skills outlined here are considered important in any consultations or interactions with patients with cancer at any stage of the disease:

- Providing supportive communication
- Identifying, and adhering to, the stated preferences of the patient in the approach to information exchange and decision-making
- Providing the opportunity for the patient to have the company of a support person during the consultation, and informing the patient about this opportunity in advance of the visit
- Showing regard and concern through the use of verbal and nonverbal behaviour that is appropriate for the patient’s age, cultural background, and preferences
- Using active empathic listening, facilitating responses by the patient
- Allowing the patient to express any concerns, fears, anxieties, or anger in a manner that aligns with the communication preferences of the patient at the time—for example, with regard to talking or crying
- Discussing embarrassing or disturbing topics in a manner that aligns with the communication preferences of the patient
- Offering access to appropriate psychosocial support services
- Communicating in understandable language
- Assessing the current understanding of the patient before providing additional information
- Explaining difficult terms and avoiding medical jargon
- When possible, using professional translation services, rather than hospital staff, volunteers, or family members
- Using strategies to aid recall and understanding
- Providing the opportunity for the patient to ask questions and seek understanding
- When appropriate, making use of simple diagrams and pictures
- Repeating and summarizing important information
- Reinforcing important information by using one or more of the following aids, if appropriate:
  - Relevant information provided in written form
  - A tape of the consultation, as needed and if wanted
  - A summary letter sent to the patient as follow-up

#### 4.1.3 Recommended Approach: Common Skills Across Critical Points of Care

In addition to the general interaction skills described in the preceding subsection, these steps are recommended when telling patients that they have cancer, a recurrence, or metastases:

- Before a discussion about diagnosis, recurrence, or metastases:
  - Ensure that the news is given in person and in a quiet, private place. Allow enough uninterrupted time. When communication in person is not possible, ensure that the patient is well supported before disclosure is made.
  - Encourage the presence of a second relevant person, if appropriate.
  - Make available other methods to convey additional information, including written materials, or videotapes or audiotapes of consultations, based on patient or family request.
- When giving information on diagnosis, recurrence, or metastases:
  - Assess the patient’s personal preferences for information and current understanding of their condition. For example, ask “Tell me, in your own words, what you understand about your condition?”
  - Briefly explain the process by which the diagnosis was reached.
  - Provide information simply and honestly, using lay terms rather than euphemisms.
  - Avoid giving the message that “nothing can be done.”
  - Clearly indicate that you will make recommendations about the range of acceptable care options, but that you are willing to be involved in ongoing care (if required), no matter what decision is made by the patient in response to the recommendations, and that patient consent is required to proceed with any care option.
  - Play an emotional and supportive role by giving the patient the opportunity to express concerns and feelings (including fears, anger, or anxieties) and to cry freely. Respond empathically to the patient’s communications.
  - Address disturbing or embarrassing topics in a manner that aligns with the patient’s preferences.
  - In concluding the discussion, summarize the main points of the consultation and assess the understanding that the patient has acquired about the information that has been communicated.
  - Ask if there is anything more that the patient would like to discuss.
  - Offer to assist in telling others about any difficult news.
  - Indicate your availability to address any questions or concerns, and arrange a further appointment to review the situation within a specified period (for example, within 24 hours to 2 weeks).
  - Assess the understanding of the patient about their prognosis.
- After a discussion of a diagnosis, recurrence, or metastases:
  - Document any information given to the patient and family members.
  - Let others, particularly the general practitioner caring for the patient, know in a timely manner the extent of the information given and your perception of the understanding of that information by the patient.
  - Ensure that there is a provider with whom the patient can debrief after the interaction.

### 4.2 How to Discuss Prognosis

#### 4.2.1 Evidence-Based Rationale

The way in which a prognosis is communicated—for example, the use of words or numbers, the discussion of survival or death as the outcome—and the time taken to explain information can influence emotional reactions in patients and decisions by those patients about treatment.

#### 4.2.2 Recommended Approach

These steps are recommended in conjunction with the general interaction skills for discussing prognosis with people with cancer:

- In offering prognostic information:
  - First, ask if the patient wants information about prognosis (for example, “I can tell you what happens to most people in your situation. Would you like me to do that?”), what the patient’s current understanding about prognosis is, and what kind of information the patient expects to receive.
  - Offer prognostic information as part of treatment decision-making before selecting and commencing treatment.
- In discussing aspects of prognosis:
  - Adhere to the patient’s stated preferences for prognostic information at that time. If or when such information is desired, the following details can be provided:
    - Staging and its  implications for prognosis
    - Chances of being  cured or that cancer will never return
    - Likely benefits  and risks of treatment
    - Average and  longest survival times, emphasizing a range rather than a single time point
- Manner of discussing prognosis:
  - Preface any prognostic estimate with its limitations:
    - Explain that you  cannot predict with certainty how individual patients will respond to the illness and its treatment.
    - Provide an  initial prognosis, based on available information, and explain how this prognosis may be revised by additional information. Suggest a time frame for when additional prognostic information is likely to be available.
  - Use mixed framing:
    - Give chances of  cure first and then chances of relapse.
  - Present information in a variety of media—words, statistics, graphs—as needed.
  - Combine verbal estimates: for example, “small chance” with numeric estimates.
  - Provide verbal explanations of survival graphs.
  - When explaining relative risk reduction, provide several examples of the calculations.
  - Use only statistical terminology such as median, hazard ratio, or relative risk, if patients are familiar with these concepts.
- In concluding the discussion:
  - Provide information that has hope-giving potential, including ongoing care that the patient will receive.
  - Provide information that is necessary for the patient to plan for the future.
  - Assess the understanding of the patient concerning personal prognosis.

### 4.3 How to Prepare Patients for Medical Procedures

#### 4.3.1 Evidence-Based Rationale

Providing patients with information about the procedure that they are about to undergo reduces emotional distress and improves psychological and physical recovery.

Practical details about the procedure (procedural information), communicated in person or through booklets, videotapes, or compact discs, may reduce psychological distress. This information can be provided by a clinician or other health professional such as a specialist nurse.

Information about the experiences that patients are likely to have before, during, and after a procedure (sensory information) decreases anxiety.

Psychosocial support before undergoing surgery reduces psychological distress.

#### 4.3.2 Recommended Approach for Preparation for Medical Procedures

These steps, in conjunction with the general interaction skills listed earlier, are recommended to adequately prepare patients for a medical procedure:

- Before the procedure:
  - Ask how much detail the patient would like to know about the procedure before you explain it.
  - Based on the stated preferences of the patient, explain why the procedure is needed and what outcome is expected.
  - Explain the possible benefits and risks involved with the procedure.
  - Provide information regarding the actual performance of the procedure, which may include these aspects:
    - Where the  procedure is likely to take place and who will perform it
    - Any tests needed  before the procedure
    - What the patient  will need to do before the procedure
    - What the patient  is likely to experience during and after the procedure
    - How any  discomfort will be managed for the patient before and after the procedure
  - Provide an opportunity for the patient to talk about concerns such as embarrassment, pain, fear, or death.
- During the procedure:
  - Offer to provide information about what will be done and how it will feel.
  - Provide the patient with the level of control that this patient feels most comfortable with (for example, asking the patient to say when he or she is ready to begin may be appropriate in some cases).
- After the procedure:
  - Encourage the patient to state needs, and reframe complaints into requests.
  - Discuss the recovery period and limitations on activities of daily living.

### 4.4 How to Discuss Treatment Options

#### 4.4.1 Evidence-Based Rationale

Appropriate and detailed information promotes understanding and increases the psychological well-being of people with cancer.

Recall of information increases when patients are provided with individualized information.

Providing a question prompt sheet to cancer patients during an initial consultation may promote their questions. It also reduces anxiety, improves recall, and shortens the consultation.

#### 4.4.2 Recommended Approach for Discussing Treatment

These steps, in conjunction with the general interaction skills listed earlier, are recommended for discussing treatment options:

- In providing information about treatment:
  - Explain to the patient, in lay terms, the treatment options that are available (including no cancer-directed treatment), and ask how much detail the patient would like to receive about each option.
  - Tailor the information to the needs and preferences of the patient for information content and detail. Include a discussion of the expected outcomes and major side effects of each treatment option.
  - Acknowledge the uncertainty that any treatment will achieve its aim. Summarize and explain the pros and cons of each treatment option.
  - Offer a variety of media providing information about treatment options: written information, decision aids, videotapes, audiotapes, or memory discs of consultations.
  - Ask whether the patient has any questions about alternative and complementary therapies.
  - Ask if the patient has concerns to express or questions to ask about the various options.
- In making decisions about treatment:
  - Explore at an early stage how the patient would like to be involved in decision-making, and adhere to the wishes expressed.
  - Be aware that the patient’s preferences may change over time. Regularly check the nature and level of participation that this patient would like to have in decision-making.
  - Ask the patient about personal values and life circumstances in relation to the treatment options.
  - Use inclusive language such as “we” and “our.”
  - Make explicit that the patient has choices to make and that personal involvement in those choices is important.
  - If the patient is unaccompanied, ask whether a discussion of treatment options with family or friends would be appreciated. Tell the patient that these individuals can have an opportunity to be involved in treatment decisions, if the patient so desires.
  - Assure the patient that there is enough time to consider treatment options. Offer to arrange a return visit when the patient has made a decision.
- In playing an emotional and supportive role:
  - Consider the specific needs related to the patient’s sex, age, and culture.
  - Offer the patient an opportunity to discuss and express concerns and feelings (including fears, anger, hopes, anxieties) and to cry freely.
  - Acknowledge individual differences in emotional impact.
  - Make your own recommendations about the range of acceptable treatments, but also express a willingness to be involved in ongoing care (if required), no matter what decision the patient makes in response to your recommendations.
- In concluding the discussion:
  - Offer assistance in telling others any difficult news about treatment choices.
- After discussing treatment options:
  - Document information given to the patient and family members.
  - Let others, particularly the general practitioner caring for the patient, know the extent of the information given and your perception of the patient’s understanding of that information.

### 4.5 Recommended Approach for Discussing Disease Progression

These steps, in conjunction with the general interaction skills described earlier, are recommended to prepare patients for progression of disease:

- Provide information openly and honestly about changes in the cancer, about treatment efficacy, and when requested, about prognosis.
- Offer the opportunity for the patient to voice concerns and goals before you discuss specific clinical decisions.
- Ask open-ended questions, such as these:
  - What concerns you most about your illness?
  - What has been most difficult about this illness for you?
  - What are your hopes and expectations and fears about the future?
  - As you think about the future, what is the most important to you?
  - Is faith (religion, spirituality) important to you in this illness? Can you describe the way in which it is important?
  - Would you like to explore these matters with someone?
- When concerns have been discussed, provide reassurance about issues such as symptom management and, when possible, the availability of expert palliative care.
- Offer the opportunity to fully explore how the patient understands palliative care. Emphasize the role of palliative care throughout illness.
- Clearly assure the patient that optimal care will be provided and that they will not be abandoned.
- In planning care:
  - Assess the abilities and willingness of the patient to be involved in decision-making. Identify a substitute decision-maker, if necessary. Check with the patient and the family members about the amount and type of information desired.
  - Discuss the usual need for greater family involvement when the awareness and communication capacities of the patient decrease with disease progression.
  - Keep the patient—and when permission has been obtained, the family—informed about issues that are most important to them, such as the likely course of the disease, symptom management, and service availability.
  - Ask about what the patient understands about their disease status, including current needs and priorities.
  - Discuss a plan for future management and monitoring, including an understanding of short- and medium-term outcome goals. Review plans and wishes for advanced care with the patient or the family, or both.
- In dealing with physical issues:
  - Introduce the notion of active treatment of symptoms and the importance to ongoing care of treating symptoms.
  - Discuss and clarify the current targets of symptom management. Actively inquire about the patient’s symptoms.
  - Provide the patient and the family with information about specific measures available for symptom relief.
- In dealing with psychological issues:
  - Offer the opportunity to discuss how the patient and the family are coping with the disease and how the reactions of others to the disease are affecting the well-being of the patient.
  - Make specific arrangements for counselling and support or information to be provided to the patient and the family by specialized psychosocial oncology staff, when appropriate and when such services are available.
- In dealing with social issues:
  - Allow an opportunity for open communication and expression by the patient of feelings and fears in relationships, and for discussions with family and friends related to dying and death.
  - Address, in a timely and sensitive manner, practical issues such as planning for care and support at home, making a will, designating power of attorney, and applying to palliative care units.

## 5. CONCLUSIONS

Clinician–patient communication is a fundamental aspect of cancer care that significantly affects the therapeutic relationship, the well-being of patients and families, treatment decision-making and compliance, and the capacity of patients and families to plan for alternative trajectories in the disease and treatment course. However, despite its importance, clinician–patient communication has been underemphasized in medical education and is often inadequate in cancer care.

Here, we have presented guidelines for communication in cancer care that were developed by an expert panel, based on a systematic review of the literature^3^,^4^,^10^, and validated by a survey of external practitioners. The results are limited by the evidence that is currently available and by the relatively low response rate from the external practitioners. Nevertheless, the recommendations received almost universal endorsement from those practitioners who did respond. We hope that these recommendations, including the key points highlighted in Table I, will be useful guideposts for the teaching and practice of communication in cancer care. Further research is needed to establish the most satisfactory methods of communication, to evaluate the role of Web-based and other technologies in communication, and to develop and evaluate more effective methods of education and training in medical communication.

## 7. CONFLICT OF INTEREST

The authors declare that no conflicts exist.

## Figures and Tables

**TABLE I:** Ten key points for clinician patient communication

Take into account the needs and preferences of patients, including those related to religion and culture, in the communication dialogue. Identify and adhere to the stated preferences of patients in the approach to information exchange and decision-making. Show regard and concern for patients by using verbal and nonverbal behaviour that is appropriate for their age, cultural background, and personal preferences. Ensure that significant news is given in person and in a quiet, private place. Allow enough uninterrupted time. Communicate information honestly, but in a way that provides room for hope and that indicates your willingness to be there for ongoing support. Consider strategies to aid recall and understanding—that is, allow questions, use diagrams, write down or tape the consultation. Allow patients to express their understanding and feelings about the information provided. Provide an opportunity for debriefing, discussion, and support after the communication of critical and upsetting information. Communicate in clear, simple terms, avoiding medical jargon. Allow for communication with patients individually and as a part of a family unit or support system.
